# Insulin Resistance, Type 1 and Type 2 Diabetes, and Related Complications 2015

**DOI:** 10.1155/2015/234135

**Published:** 2015-07-28

**Authors:** Joseph Fomusi Ndisang, Sharad Rastogi, Alfredo Vannacci

**Affiliations:** ^1^Department of Physiology, University of Saskatchewan College of Medicine, 107 Wiggins Road, Saskatoon, SK, Canada S7N 5E5; ^2^The Medical Affairs Company, Cardiovascular Division, 43229 Dequindre Road, Troy, MI 48085, USA; ^3^Department of Neurosciences, Psychology, Drug Research and Child Health, Section of Pharmacology and Toxicology, University of Florence, Viale Pieraccini 6, 50139 Florence, Italy

The dramatic increase in obesity and diabetes worldwide poses a huge socioeconomic burden to healthcare systems. In type 1 diabetes, autoimmune-mediated destruction of pancreatic beta-cell results in insulin deficiency [1]. Obesity is one of the major causes of type 2 diabetes [1–3]. In type 2 diabetes, a combination of peripheral insulin resistance and aberrant production of insulin are amongst the paradox commonly encountered in the pathogenesis of the disease [1–3]. However, both forms of diabetes are characterized by elevated inflammation/oxidative stress, glucotoxicity, lipotoxicity, endoplasmic reticulum-induced stress with increased apoptosis and necrosis that ultimately leads to destruction loss of beta cells, and related complications including cardiomyopathy, nephropathy, neuropathy, and hepatopathy [1, 4–7]. Although insulin resistance has traditionally been associated with type 2 diabetes, recent evidence suggests that insulin resistance in type 1 diabetes is increasing [8–10]; therefore, novel mechanistic approaches deciphering insulin resistance are needed.

The etiology of insulin resistance is complicated and several factors are implicated, so deciphering this multifaceted disease remains challenging, although a wide body of evidence suggests that oxidative stress, inflammation, genetic, habitual, environmental, and epigenetic factors may be involved [1, 11]. Thus, further research is needed for more in-depth and comprehensive understanding of the pathophysiology of insulin resistance in both type 1 and type 2 diabetes, and especially in situations where diabetes is comorbid with other chronic conditions such as obesity and hypertension.

This special issue will showcase a broad spectrum research and review papers addressing thematic problems associated with insulin resistance, type 1 diabetes, type 2 diabetes, and related complications. To underscore the role of insulin resistance in children, M. P. van der Aa et al. wrote a research article on the prevalence and incidence of childhood insulin resistance, while S.-H. Nam and coworkers investigated the effects of cardioankle vascular index on metabolic syndrome, a multifactorial condition characterized by insulin resistance, dyslipidemia, hyperglycemia, hypertension, and other factors. Similarly, R. Burrows et al. gave further insights for diagnosing metabolic syndrome in adolescents in a research article. In another related research article, M. Fabregat et al. investigated the genetic profile of human leukocyte antigen (HLA) alleles and non-HLA in type 2 diabetes, while P. Tiwari wrote a systematic review about the current therapeutic strategies for the management of diabetes. Given that diabetes and hypertension are characterized by elevated inflammation/oxidative stress [4, 12–14] and these two pathophysiological driving forces are implicated in many cardiac complications [4, 12–14], J. Klen et al. investigated the role of NLRP3 inflammasome polymorphism in type 2 diabetes, shedding novel insights on the role of NLRP3 polymorphism on myocardial infarction, a macrovascular complication of diabetes. Similarly, H. Al-Safar et al. investigated the role of genetic polymorphisms on transcription-factor-7-like 2 and peroxisome proliferator-activated receptors-*γ*2 in type 2 diabetes and obesity, while M. Guclu et al. wrote a research article about the effects of combination therapy with rosiglitazone and insulin on inflammatory insults in patients with type 1 diabetes. In another related research article, Z. Yida et al. reported that cotreatment with the cholesterol lowering drug simvastatin and edible bird's nest (EBN), a traditional product commonly consumed in Asia for its nutritional value, improved insulin signaling in a rat model of high-fat diet-induced insulin resistance. Consistently, in another related study, A. Ferreira-Hermosillo et al. gave further insights on the role of inflammatory cytokines in patients with metabolic syndrome. Within the same theme of insulin resistance and metabolic syndrome, R. Adela and S. K. Banerjee wrote a review article to underscore the role of growth-differentiation-factor-15 in diabetes and related cardiovascular diseases, whereas J. Zhang et al. gave further insights on obesity and type 2 diabetes in a research article. By the same token, T.-Y. Chuang et al. reported the effects of microRNA-223 on insulin resistance by studying the adipose tissue. Finally, S. Riaz wrote an article giving novel insights on the role of Vitamin B1 on biomarkers of diabetes type 2 diabetes.

Collectively, the articles featured in this special issue cover a wide spectrum of thematic issues of great interest and would benefit a wide audience.

*Joseph Fomusi Ndisang* *Joseph Fomusi Ndisang* *Sharad Rastogi* *Sharad Rastogi* *Alfredo Vannacci* *Alfredo Vannacci*
